# It's Not Just What's Said, But How: The Disruption of Prosody Perception in Cognitive Decline and Hearing Loss

**DOI:** 10.1002/alz70856_107723

**Published:** 2026-01-09

**Authors:** Deepashri Agrawal, Palash K Malo, Shridhar Krishnamurti

**Affiliations:** ^1^ Centre for Brain Research, Bangalore, Karnataka, India; ^2^ Centre for Brain Research, Indian Institute of Science, Bangalore, Karnataka, India; ^3^ Professor of Audiology, Auburn University, Auburn, AL, USA

## Abstract

**Background:**

Age related hearing loss (HL) is highly prevalent in older adults and is frequently exacerbated by cognitive decline, such as Alzheimer's disease (AD). While speech communication primarily focusses on segmental content, the prosody‐ i.e suprasegmental aspects encompassing rhythm, stress and intonation play a critical role in conveying meaning, emotion and syntactic structure. As prosody relies on both auditory and cognitive mechanisms, both of which most of the time are significantly impaired in AD. This study investigates how cognitive impairment affects prosody perception in older adults with HL_AD compared to individuals with HL but no AD.

**Method:**

Participants were grouped into two: 1) six older adults (65–85 years) with HL‐AD, and 2) six older adults with HL but no AD. The Vocalic Sensitivity Test (VST), which measures prosodic elements such as word stress, grammatical function, emotional prosody, and intonation, was used. The VST involved listening to time‐compressed speech and identifying prosodic features. Stimuli were delivered through TDH‐39 earphones, and responses were recorded on a laptop with dual raters to ensure reliability. The Mann‐Whitney U‐test was used to check for the differences in VST scores between the study groups. Median and interquartile range (IQR) are reported, and the statistical significance was based on 10000 samples using the Monte Carlo method at 5% level of significance.

**Result:**

Monte Carlo simulated significance levels showed a significant difference between the groups for intonation (control: median (IQR) 8.00 (4.75); HL‐AD: 3.50 (2.50); *p* =  0.032), word stress (control: 8.00 (5.75); HL‐AD: 3.50 (2.00); *p* =  0.044), grammatical function (control: 6.50 (3.50); HL‐AD: 3.00 (2.75); *p* =  0.025) and total VST score (control: 27.00 (12.25); HL‐AD: 14.50 (3.75); *p* =  0.033). However, there was no significant difference for emotional prosody (control: 4.50 (2.75); HL‐AD: 3.50 (1.25); *p* =  0.276).

**Conclusion:**

This study reveals that older adults with AD_HL experience considerable difficulty distinguishing prosodic aspects of speech, particularly in word stress, grammatical function, and intonation. Thus improving prosodic perception along with auditory and cognitive abilities is crucial for better communication outcomes, social interaction, and speech comprehension, all of which would improve the quality of life.

